# Awareness, Knowledge and Attitude towards ‘Superfood’ Kale and Its Health Benefits among Arab Adults

**DOI:** 10.3390/nu14020245

**Published:** 2022-01-07

**Authors:** Hanan A. Alfawaz, Kaiser Wani, Haya Alrakayan, Abdullah M. Alnaami, Nasser M. Al-Daghri

**Affiliations:** 1 Department of Food Science & Nutrition, College of Food Science & Agriculture, King Saud University, Riyadh 11495, Saudi Arabia; halfawaz@ksu.edu.sa (H.A.A.); alrakayan.h@gmail.com (H.A.); 2 Biochemistry Department, College of Science, King Saud University, Riyadh 11451, Saudi Arabia; kwani@ksu.edu.sa (K.W.); aalnaami@ksu.edu.sa (A.M.A.)

**Keywords:** kale, superfood, obesity, Saudi Arabia, nutrient-dense

## Abstract

This cross-sectional online survey aimed to determine the awareness of Arab adults on the benefits of consuming nutrient-dense foods, such as kale. A total of 1200 respondents completed the survey. The questionnaire included questions related to socio-economic information, e.g., whether the participants have consumed kale, if they observed any health effects, and 13 other questions to test their knowledge on this superfood. Only 276 (23%) of the participants had previously consumed kale, with 64.5% reporting favorable health outcomes, the most common of which was weight reduction, and only 17.8% reporting side effects, such as constipation and gastrointestinal irritation. From the 13 kale knowledge questions, the average total knowledge score, scaled from 0 to 10, was 3.5 and 3.7 for males and females, respectively. The regression analysis revealed that age, income, and educational status were significant contributors for predicting better knowledge scores, as older individuals with a higher income and higher education scored higher (odds ratio of 2.96, 2.00 and 4.58, respectively). To summarize, there is a dearth of awareness about kale and its health benefits in Saudi Arabia. Kale should be promoted as a super food in all segments, particularly among the younger, lower-income, and less-educated sections of the population.

## 1. Introduction

Obesity is a global issue, as nearly half of the world’s population aged 18 years or older is either overweight (39%) or obese (13%) [1]. One of the leading causes of obesity in the recent decades has been the increasing consumption of calorie-dense foods, which are rich in refined sugars, carbohydrates, and fats [2,3]. These foods are highly processed, manufactured from a few key ingredients, and, hence, devoid of the necessary nutritional components, resulting in not only an excess of sugars and fats in the body, but also increasing micronutrient deficiencies [4]. This nutritional transition over the last few decades has been an important factor in the ever-increasing prevalence of non-communicable diseases, such as type 2 diabetes, stroke, hypertension, dyslipidemia, osteoarthritis, etc., which, together, result in an estimated 36 million deaths each year [5,6,7].

To counter the menace of obesity, several approaches have been applied by both government and private organizations, including interventions to change physical activity levels, dietary habits and/or social behavior. Our group at the Chair for Biomarkers of Chronic Diseases (CBCD) has been facilitating interventions in vitamin D supplementation, physical activity, and dietary changes over the last 12 years, in order to promote healthy lifestyles in the Saudi population [8,9,10,11,12,13]. Over the past few decades, the food habits of the Saudi Arabian population have transitioned from a traditional Arab diet to a westernized calorie-dense diet of fast foods, carbonated drinks, desserts, etc.; this has been observed due to the higher incomes and rapid urbanization [14,15]. One of the important impacts of such dietary trends in a society, apart from the increased incidence of non-communicable diseases, is the increased deficiencies in critical nutrients, especially micronutrients. More than two billion people worldwide suffer from micronutrient malnutrition, also known as hidden hunger [16,17]. The results of our study in 2019 highlighted the low intake of important micronutrients, such as vitamin A, vitamin E, thiamine, folate, potassium, etc., in the routine Saudi diet [18]. To address this, dietary interventions, such as the introduction and promotion of low-calorie, low-fat, and nutrient-dense foods, need to be implemented. Kale (*Brassica oleracea* v. *acephala*) is one superfood that has long been cultivated as a health crop, but its entire range of benefits and its composition have only just begun to be investigated in the western world.

Kale, a member of the *Brassicaceae* vegetable family, is a cabbage-like plant characterized by non-heading green leaves [19]. It is a cool seasonal plant grown during early spring, with evidence showing that its use as a food crop dates back to 2000 B.C. in Eastern Mediterranean regions, but it has only gained attention from the scientific community in recent years [20,21]. Because of their nutrient-rich composition, these green vegetable crops may supply much-needed nutrients to consumers, and are frequently marketed as health superfoods. Kale, mostly eaten raw in the form of salads, contains a variety of micronutrients, including vitamins and minerals, antioxidants, carotenoids, glucosinolates, and polyphenols, which are beneficial to human health [22]. Kale is high in many vitamins (A, K, C, and folate), important minerals (potassium, calcium, and magnesium), and dietary fiber [23]. Kale is also a good source of prebiotic carbohydrates and phytochemicals, such as folic acid, riboflavin, carotenes, and others that serve as antioxidants, capturing free radicals and aiding in the reduction in inflammation [24]. Owing to these properties, kale has become widely known as a medicinal food source to treat bowel ailments, obesity, malnutrition problems, and chronic diseases, such as cardiovascular disease and cancer; to reduce DNA damage and enhance vitamin C, carotene, and erythrocyte glutathione peroxidase activity; to increase cytochrome CYP1A2 activity, which is important for metabolism [25,26,27]. Nonetheless, while being extremely nutrient dense, kale has a relatively low calorie content (36–98 kcal/100 g) [28]. This has made kale one of the superfoods that should be promoted as a healthy alternative to already popular calorie-dense food products.

Unfortunately, in the Saudi population, the superfood “kale” seems to be almost unknown, as no single article with the keywords “kale” and “Saudi Arabia” could be retrieved (as of 2 January 2021). Our team intends to conduct a promotion drive in this population, regarding the beneficial effects of kale consumption. At the same time, we have applied for a research fund for an intervention study to evaluate the effects of consuming a dietary supplement, freeze-dried kale, on metabolic health, microbiota composition, and inflammatory insults. However, prior to that, it is important to investigate the current knowledge of kale and its health benefits according to socio-economic strata, as this may allow potential intervention targets to be identified. Thus, in this study, we aimed to investigate the knowledge of kale and its consumption benefits among different socio-economic categories in the Saudi population.

## 2. Materials and Methods

### 2.1. Study Design and Participants

This cross-sectional online survey was designed to study the knowledge and awareness of kale consumption among Saudi adults. This survey was conducted from 20 October to 8 December 2020. A questionnaire was designed and cascaded to all employees through their registered institutional e-mails in the KSU database. The questionnaire link was also distributed on social media apps such as WhatsApp groups and Twitter handles. One response per ID was allowed to ensure no duplication of data. The inclusion criterion was all Saudi adults, while the exclusion criteria included non-Saudi residents living in Saudi Arabia.

### 2.2. Questionnaire

A test study (*N* = 100 participants) was performed to ascertain the questionnaire’s reliability and validity. To guarantee the clarity of the questions, content and face validity tests were conducted and several changes were made to improve the reliability and scientific value of the data to be collected. The Cronbach’s coefficient reliability test yielded more than 70% for each component of the questionnaire with a Cronbach’s alpha of 0.902. The questionnaire included a cover letter in Arabic and English. The final version was uploaded to an internet link and distributed through e-mail and social media.

The questionnaire consisted of the following three sections:

1. The first section asked about the participants’ socio-demographic information, which included age, sex, marital status, family income, educational qualification, etc.
2. In the second section, 13 questions, which determined the kale nutritional value knowledge among Saudi adults, were asked to the study participants. The questions included information about kale being a food with high calorie content, with high nutritional value, with immune boosting properties, rich in fiber, rich in vitamin C, and low in fats. This section also included questions about benefits of kale consumption in health conditions such as constipation, digestive problems, cancer, inflammation, oxidative stress, and chronic diseases. The study participants were also asked whether or not cooking kale decreases its nutritional and health benefits.
3. In the final portion, research participants were asked if they had ever consumed kale previously, and if so, what positive or negative health impacts they experienced. The questions included information about how many times a week the participants were consuming kale and if there were any health effects, such as weight loss, decrease in appetite, improved bowel movements, etc., felt by the participants during this time, or if they experienced any side effects, such as constipation, bloating, bowel irritability, etc. Additionally, the mode of consumption of kale was also asked in this section, such as consumed fresh, cooked, as supplements, or as the commercially available powdered form.

### 2.3. Data Analysis

SPSS version 23.0 (Chicago, IL, USA) was used for the data analysis. Continuous and categorical variables were depicted as mean ± standard errors (SEM) and frequencies (percentages), respectively. Chi-square and independent t-tests were employed to compare categorical and continuous data, respectively. The proportion of participants who chose the correct answers to the questions in the kale knowledge segment was shown as N (percentage among total). The overall kale knowledge of each participant was scored as 0–10, with 0 for those who did not answer a single question correctly and 10 for those who answered all 13 questions correctly. This score was used to determine the average kale knowledge in each group. The study participants were stratified into tertiles based on this kale knowledge score, with tertile 1 and tertile 3 being the lowest and highest kale knowledge, respectively. To investigate determinants of increased kale knowledge among groups, a multinomial logistic regression analysis was performed using kale knowledge tertiles as the dependent variable and socio-demographic statuses as the independent variables. This was performed for all subjects, then separately for both sexes. *p*-value was considered significant at *p* < 0.05. Microsoft Excel was used to plot the figures.

## 3. Results

### 3.1. Characteristics of the Study Participants

The data regarding the characteristics of the study participants are presented in Table 1. One thousand two hundred Saudi adults participated in the survey, out of which 39.7% were males and 60.3% were females. Half of the participants (53.7%) represented the age group of 26–35 years, and about 1/4 of the study participants represented the age groups 18–25, 36–45, and >45 years each. There was no statistical difference in the age distribution between the sexes. Nearly half of the participants (54.8%) were unmarried, while 8.2% of the study participants were divorced. The distribution of marital status between the males and females was statistically similar. A large proportion of the participants (71.2% of the males and 60.8% of the females) had an average monthly income in the middle to above-average range (SAR 9001–21000). Similarly, the data on the educational status of the participants showed that a large proportion (62.2% of the males and 71.2% of the females) were either educated up to graduation level or were post-graduates.

### 3.2. Knowledge about Benefits of Kale Consumption in the Study Participants

The knowledge about kale and its health effects was tested by asking the participants 13 questions regarding the properties of kale and its consumption, and the data are presented in Table 2. On average, almost 60% of the participants answered correctly when asked about kale being a food with a high calorie content, with a high nutritional value, with immune boosting properties, rich in fiber, and low in fats. However, there seems to be less knowledge about the benefits of kale consumption on health conditions, such as constipation (11.5%), digestive problems (13.7%), cancer (17.2%), inflammation (19.0%), oxidative stress (29.8%), etc. Additionally, only 16.1% of all the participants knew that cooking kale decreases its nutritional value. Moreover, an overall “kale knowledge score” was devised, where those who answered all 13 questions correctly were scaled as “10” and those who answered none correctly were scaled as “0”, and 3.6, 3.5 and 3.7 as the overall kale knowledge scores depict the average knowledge score in all the subjects, in males and in females, respectively. The overall data from the kale questionnaire are tabulated separately and presented in Supplementary Table S1.

**Figure 1:**
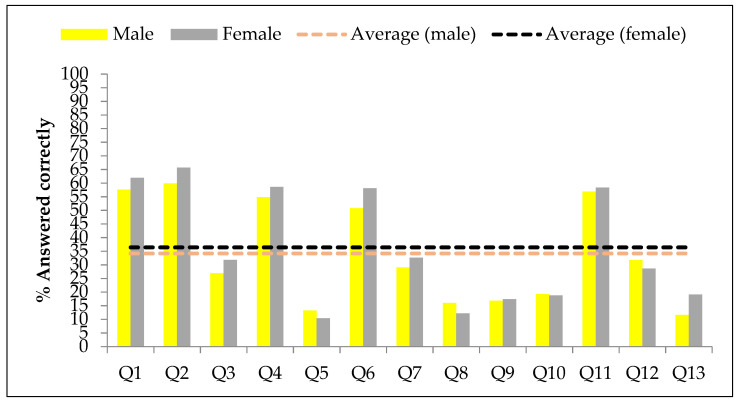
A bar graph depicting the knowledge about benefits of kale consumption among the study participants. The dotted line shows the average in both sexes.

The proportion of participants who answered the kale knowledge questions correctly is plotted as a bar graph in Figure 1.

### 3.3. Kale Knowledge Score in Study Participants Divided as Per Socio-Demographic Variables

The knowledge regarding the benefits of kale consumption was checked in the study participants, who were divided as per their socio-demographic variables, and the results are summarized in Table 3. The total knowledge score (scaled as 0–10) in the study participants was divided into tertiles, with tertile 1 and tertile 3 having the lowest and highest kale knowledge scores, respectively, and a regression analysis was run. For all the subjects and the females only, age, family income, and educational status were all significant contributors for predicting better knowledge scores, as the older participants with a higher income and higher educational qualification scored higher compared to the younger participants with a lower income and lower educational qualification. In males, a higher educational qualification was the sole significant contributor among the socio-demographic variables, with an odds ratio of 2.66 (*p* < 0.05) in post-graduates compared to those who have only studied to secondary level.

**Figure 2:**
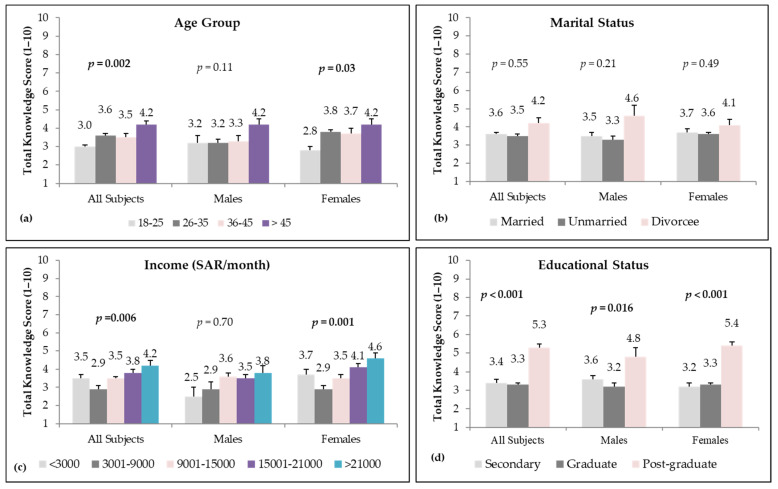
A bar graph depicting the kale knowledge scores in study participants divided according to (**a**) age group, (**b**) marital status, (**c**) income and (**d**) educational status. The error bar shows the standard error of the mean.

The kale knowledge scores for the groups according to socio-demographic status are plotted on the bar graphs in Figure 2, which show that age group, monthly income, and educational status were significant contributors in obtaining higher scores.

### 3.4. Consumption of Kale in Study Participants and Its Health Effects

The study participants were asked whether they have ever consumed kale and what the positive or negative health effects were. These data are summarized in Table 4. When asked whether they have heard about kale, 53% (N = 384) of the females and 42.2% (N = 201) of the males responded affirmatively. Only 26.1% of the females and 18.3% of the males reported having consumed kale before. Among those who had consumed kale before, 60.8% of the females and 71.3% of the males reported positive health effects, while only 16.9% and 19.5% of the females and males, respectively, reported side effects. The most common positive health effect reported was weight loss, followed by a decrease in appetite, improved bowel movement, and a boost in energy levels. The most common side effects reported were constipation, bloating, and bowel irritability.

To summarize, there is a low level of awareness about the health benefits of nutrient-dense food, such as kale, in Saudi adults. This low awareness level is worse in some sections of the society, such as the younger population, those with less income, and the less educated. Additionally, the results showed that a low percentage of Saudi adults have consumed kale. However, the majority of those who had consumed kale reported beneficial health effects, such as weight loss and improved bowel movement.

## 4. Discussion

The current survey-based study examined the Saudi adult population’s knowledge and awareness of kale and its health benefits according to their socio-economic strata, and pointed out a need to introduce and promote a dietary shift from a calorie-dense, highly processed diet to a nutrient-dense, natural-ingredient-based diet. The study highlights the sub-populations in which the knowledge of superfoods, such as kale, is especially low, and that promotion drive targeting in such sections needs to be intensified. The study also enlists a low percentage of participants who have used kale in their diets, the majority of whom have reported weight loss as a health benefit.

Over the last few decades, a nutritional transition in Saudi Arabia has been an important factor in the ever-increasing prevalence of non-communicable diseases, such as obesity, type 2 diabetes, dyslipidemia and cardiovascular disease [29,30]. At the same time, this trend has increased the hidden hunger characterized by micronutrient deficiencies [31,32]. The introduction of nutrient-dense natural food items, such as kale, in the regular diet of Saudi Arabians may be an essential step towards correcting this nutrient deficiency in the population. Kale, a green leafy brassica vegetable related to broccoli, cabbage, and cauliflower, was traditionally known in the Netherlands, Scotland, and Northern Germany, and is only recently quickly becoming a popular specialty crop in the United States, particularly in the southern states of North and South Carolina, due to its high nutritional value [33]. We devised a 0–10 kale knowledge scale in this study, based on the 13 questions asked, related to kale and the health benefits of its consumption. The overall average knowledge score obtained by the participants was 3.5 and 3.7 among the males and females, respectively, which indicated a low level of awareness of kale and its health benefits in the adult Saudi population.

An average level of awareness was observed in the participants when asked about the properties of kale, such as the calorie content, nutritional value, immune boosting properties, high fiber content, and low fat content (about 60% of the participants answered these questions correctly). Kale is a nutritional powerhouse. According to food composition databases released by the United States Department of Agriculture (USDA), one cup of kale provides around 4.3 g of protein, 8.8 g of carbohydrates, 3.6 g of which are dietary fibers (12.9% of the daily requirement), only 0.93 g of total fats, and 49 calories [34]. This accounts for about 8%, 12.9%, only 1.4%, and 2% of the average adult daily requirements of proteins, dietary fibers, fats, and energy (Supplementary Table S2). This implies that the consumer’s health may be substantially enhanced by researching the nutritional content of kale, boosting the intake of nutrient-rich variants, and enhancing nutrient bioavailability [35,36]. The nutritional knowledge about superfoods, such as kale, is not widely known, and the literature about the level of awareness regarding kale and its health benefits in different populations is scarce; however, a new found interest in health and diet, particularly in health-conscious people, might have led to the average level of knowledge about kale in our participants. At the same time though, this knowledge is not reflected in practice, as only 23% of the respondents reported having ever used kale. A public awareness campaign on nutrient-dense vegetables, such as kale, would be required in this population, in order to include these foods in the regular Saudi diet, while also encouraging people to reap their health benefits.

When asked about the specific benefits of kale consumption, such as it being rich in micronutrients, such as vitamin C; health benefits, such as its antioxidant, anti-inflammatory, and anti-carcinogenic properties, and the fact that it lowers the risk of chronic diseases; side effects, such as constipation and digestive problems, only 21.7 percent of the participants answered correctly. To encourage people to try this superfood, it is necessary to educate them about “micronutrient deficiency”, or “hidden hunger”, which refers to the insufficient intake or inefficient biological utilization of micronutrients as a result of a calorie-dense, but nutrient-deficient diet [37,38]. Awareness about the micronutrient content of kale and its health impact will eventually help consumers to make the right choice when it comes to a healthy nutritious diet. The micronutrient content of kale is very high, and, thus, it has been listed in the top 10 superfoods by the USDA [39]. Kale has high amounts of the vitamins K, C, A, E, B6, folate, and riboflavin, and one serving, equivalent to one cup of raw kale, can satisfy a large portion of the average recommended daily intake (RDI) for these vitamins (Supplementary Table S2) [39]. Furthermore, it is a rich source of essential minerals, such as potassium, manganese, calcium, iron, magnesium, and phosphorous, which are required in small amounts by humans for a variety of functions [28,40,41].

The health benefits of consuming kale, such as its antioxidant, anti-inflammation and anti-carcinogenic properties, should be highlighted in awareness programs, in order to promote and introduce kale into the Saudi diet. Kale contains antioxidant and anti-inflammatory components, such as polyphenols, carotenoids, and glucosinolates, along with vitamin C and E, which can help protect against free radicals and reactive oxygen species (ROS) [42,43]. The anti-carcinogenic activity has been attributed to the decomposition products of glucosinolates present in kale, such as indole-3-carbinol, a decomposition product of glucobrassicin [44]. Kale has been reported to possess antigenotoxic potential, preventing genetic damage in a cell, which may to lead to mutations and cancer [45]; it has also been reported to possess antiproliferative potential against certain carcinomas [46]. Another hydrolysis product of glucoiberin, called sulforaphane, that is present in kale has been associated with the inhibition of antibiotic-resistant strains of *Helicobacter pylori*, and, thus, aids in reducing the risk of stomach tumors [47]. Additionally, kale has been associated with enhancing gut microbiota diversity, which not only plays an important role in metabolic and neurological health, but also aids in reducing endotoxemia-mediated, obesity-associated low-grade inflammation [28,48].

The present study also highlighted the need to target younger, less-educated and lower-income sections of the population with awareness programs about the dietary shift to a healthier diet and the inclusion of nutrient-dense foods, such as kale, in the regular diet. The average knowledge and awareness score about kale was significantly lower in these sections of the Saudi population, as calculated by the odds ratio in the regression analysis performed in this study. A number of studies have reported a dietary shift in the Saudi population in the past, from a traditional Arab diet to a calorie-dense western diet, characterized by the dominance of processed foods and sugary beverages, especially in young adults, making them a high-risk group for failing to meet the average dietary recommendations, particularly for micronutrients [49,50,51]. The socio-economic status of individuals, including education and income, at the same time, similarly to in this study, has also been studied as an established determinant when it comes to its influence on healthy lifestyle behavior, including dietary choices [52,53]. An intervention of educational programs on dietary choices and the inclusion of nutrient-dense food items, such as kale, in the regular diet needs to be launched, especially targeting the younger, lower-income and less-educated sections of the Saudi population. If properly investigated, biofortified through biotechnology and breeding programs, and promoted through awareness programs, superfoods, such as kale, have the potential to eliminate calorie starvation, micronutrient deficiencies, and chronic diseases throughout the world.

The current study’s methodological strength, apart from having an adequate sample size, was that it was the first study to assess kale awareness, attitude, and knowledge in the Saudi population. The authors, however, would like to point out some limitations of the study. Being survey-based research, this study may just provide a snapshot of the level of awareness about and attitude towards superfoods, such as kale, in the population studied, and may not reflect the actual usage of kale in the Saudi diet, since strategies such as the use of food frequency questionnaires were not implemented in this study. The study may also not reflect the actual clinical states and benefits of consuming superfoods, such as kale, including weight reduction reported by the respondents who had used kale, since no clinical measurements were performed in this study. Being a questionnaire-based survey, the investigators had no control in choosing the sample size and characteristics, as clear by the disproportion in sample size between the sexes. Though this does not influence the results overall, as the knowledge about and attitude towards kale were reported separately for the sexes here, the investigators suggest that future research should conduct an interventional study in which kale supplements are tested for beneficial health effects, such as weight loss, etc. Having pointed out the limitations, however, the current study was designed to investigate the effect of the socio-economic status of adult Saudis on the awareness and attitudes towards kale before inclusion in several local awareness programs and lifestyle intervention studies previously launched for populations at risk [11,12,13,18,54]. This study also provides a glimpse into the low awareness of nutrient-dense foods, such as kale, and the need to introduce it into Saudi dietary habits. The study may guide policy makers and authorities to implement steps for the awareness of the health benefits of nutrient-dense foods, such as kale, in all sections of a population, especially in younger, low-income and less-educated sections. This has been added in the revised text.

## 5. Conclusions

There is a lack of knowledge in Saudi Arabia regarding kale and its benefits to health. This study suggested that the awareness about this superfood, though low in all socio-economic sections, is especially low in younger, low-income and less-educated populations. This study also suggested significant beneficial health effects of kale consumption, such as weight loss, as anecdotally reported by a high proportion of respondents who tried this superfood.

## Figures and Tables

**Table 1:** Socio-demographic characteristics of the study subjects.

Parameters	All	Males	Females
N	1200	476	724
Age Group (Years)			
18–25	174 (14.5)	61 (12.8)	113 (15.6)
26–35	644 (53.7)	217 (45.6)	427 (59)
36–45	200 (16.6)	105 (22.1)	95 (13.1)
>45	182 (15.2)	93 (19.5)	89 (12.3)
Marital Status			
Married	445 (37.1)	175 (36.8)	270 (37.3)
Unmarried	657 (54.8)	271 (56.9)	386 (53.3)
Divorced	98 (8.2)	30 (6.3)	68 (9.4)
Family Income (SAR/Month)			
<3000	138 (11.5)	22 (4.6)	116 (16)
3001–9000	161 (13.4)	57 (12)	104 (14.4)
9001–15,000	464 (38.7)	186 (39.1)	278 (38.4)
15,001–21,000	315 (26.3)	153 (32.1)	162 (22.4)
>21,000	122 (10.2)	58 (12.2)	64 (8.8)
Educational Status			
Secondary	345 (28.8)	180 (37.8)	165 (22.8)
Graduate	691 (57.6)	262 (55)	429 (59.3)
Post-graduate	164 (13.7)	34 (7.1)	130 (18)

Note: the data are presented as frequency (%).

**Table 2:** Kale knowledge score for the study participants.

Kale Knowledge Questionnaire	All (1200)	Male (476)	Female (724)	*p*
Is kale considered a high calorie food?	60.2	57.6	61.9	0.15
Is kale considered a high nutritional value food?	63.4	59.9	65.7	0.11
Is kale considered a food with antioxidant properties?	29.8	26.9	31.8	0.07
Is kale considered a food with immune boosting properties?	57.1	54.8	58.6	0.31
Does excessive eating of kale cause constipation?	11.5	13.2	10.4	0.06
Is kale considered a food rich in fibers?	55.3	50.8	58.1	0.02
Is kale considered a food rich in vitamin C?	31.2	29.0	32.6	0.37
Is kale consumption considered bad for people suffering from digestive problems?	13.7	16.0	12.2	0.05
Is kale considered a food with anti-carcinogenic properties?	17.2	16.8	17.4	0.88
Is kale considered a food with anti-inflammatory properties?	19.0	19.3	18.8	0.92
Is kale considered a food high in fats?	57.8	56.9	58.3	0.19
Does kale play a role in controlling chronic diseases?	29.8	31.7	28.6	0.47
Does the nutritional value of kale increase after cooking?	16.1	11.6	19.1	<0.01
Total Knowledge Score	3.6 ± 0.1	3.5 ± 0.1	3.7 ± 0.1	0.12

Note: The data are presented as % of participants who answered correctly. Kale knowledge score was devised such that if all 13 questions were answered correctly it was correspondingly scaled as “10”, while those who had no correct answers at all were scaled as “0”. The difference between males and females was calculated using chi-square test and independent samples t-test for proportions and continuous normal variables, respectively.

**Table 3:** Knowledge scores according to socio-demographic characteristics.

Parameters	All Subjects (1200)				
	Tertile 1 (400)	Tertile 2 (400)	Tertile 3 (400)	Tertile3 vs. Tertile1	
Total Knowledge Score	0.5 ± 0.0	3.5 ± 0.1	6.8 ± 0.1	OR (95% CI)	*p*-value
Age Group (Years)					
18–25 (174)	43.1	32.8	24.1	Reference	0.002
26–35 (644)	32.1	35.4	32.5	1.80 (1.2, 2.8) **	
36–45 (200)	35.5	29	35.5	1.79 (1.1, 2.9) *	
>45 (182)	25.8	31.3	42.9	2.96 (1.8, 5.0) **	
Marital Status					
Married (445)	33.7	33	33.3	Reference	0.55
Unmarried (657)	33.9	33.8	32.3	0.96 (0.7, 1.3)	
Divorced (98)	27.6	31.6	40.8	1.50 (0.9, 2.6)	
Family Income (SAR/Month)					
<3000 (138)	39.1	31.2	29.7	0.80 (0.5, 1.3)	0.006
3001–9000 (161)	41	34.8	24.2	0.63 (0.4, 0.9) *	
9001–15,000 (464)	35.1	31.7	33.2	Reference	
15,001–21,000 (315)	28.3	35.9	35.9	1.34 (0.9, 1.9)	
>21,000 (122)	23	33.6	43.4	2.00 (1.2, 3.3) **	
Education Level					
Secondary (345)	34.8	34.2	31	Reference	<0.001
Graduate (691)	37.2	34	28.8	0.87 (0.6, 1.2)	
Post-graduate (164)	14	28.7	57.3	4.58 (2.7, 7.7) **	
Parameters	Males (476)				
	Tertile 1 (159)	Tertile 2 (158)	Tertile 3 (159)	Tertile 3 vs. Tertile 1	
Total Knowledge Score	0.2 ± 0.0	2.8 ± 0.1	6.6 ± 0.1	OR (95% C.I.)	*p*-value
Age Group (Years)					
18–25 (61)	36.1	34.4	29.5	Reference	0.11
26–35 (217)	33.6	37.3	29	1.05 (0.5, 2.1)	
36–45 (105)	38.1	27.6	34.3	1.10 (0.5, 2.3)	
>45 (93)	25.8	29	45.2	2.14 (0.9, 4.7)	
Marital Status					
Married (175)	33.1	33.7	33.1	Reference	0.21
Unmarried (271)	34.3	34.3	31.4	0.91 (0.6, 1.5)	
Divorced (30)	26.7	20	53.3	2.00 (0.8, 5.0)	
Family Income (SAR/Month)					
<3000 (22)	45.5	31.8	22.7	0.45 (0.1, 1.4)	0.70
3001–9000 (57)	38.6	36.8	24.6	0.59 (0.3, 1.3)	
9001–15,000 (186)	31.7	33.3	34.9	Reference	
15,001–21,000 (153)	32	34	34	0.96 (0.6, 1.6)	
>21,000 (58)	32.8	27.6	39.7	1.10 (0.5, 2.2)	
Education Level					
Secondary (180)	32.2	31.1	36.7	Reference	0.016
Graduate (262)	35.9	35.5	28.6	0.72 (0.5, 1.1)	
Post-graduate (34)	20.6	26.5	52.9	2.66 (1.1, 7.0) *	
Parameters	Females (724)				
	Tertile 1 (241)	Tertile 2 (241)	Tertile 3 (242)	Tertile 3 vs. Tertile 1	
Total Knowledge score	0.7 ± 0.1	3.9 ± 0.1	6.9 ± 0.1	OR (95% CI)	*p*-value
Age Group (Years)					
18–25 (113)	46	31	23	Reference	0.03
26–35 (427)	30.9	35.4	33.7	2.18 (1.3, 3.7) **	
36–45 (95)	33.7	29.5	36.8	2.19 (1.1, 4.3) *	
>45 (89)	28.1	30.3	41.6	2.96 (1.5, 5.9) **	
Marital Status					
Married (270)	35.2	30.4	34.4	Reference	0.49
Unmarried (386)	32.9	35.5	31.6	0.98 (0.7, 1.4)	
Divorced (68)	27.9	32.4	39.7	1.45 (0.8, 2.8)	
Family Income (SAR/Month)					
<3000 (116)	37.1	25.9	37.1	1.18 (0.7, 1.9)	0.001
3001–9000 (104)	42.3	31.7	26	0.73 (0.4, 1.3)	
9001–15,000 (278)	37.1	31.7	31.3	Reference	
15,001–21,000 (162)	26.5	37.7	35.8	1.59 (0.9, 2.6)	
>21,000 (64)	12.5	45.3	42.2	3.99 (1.7, 9.2) **	
Education Level					
Secondary (165)	40.6	34.5	24.8	Reference	<0.001
Graduate (429)	36.8	34	29.1	1.29 (0.8, 2.0)	
Post-graduate (130)	12.3	29.2	58.5	7.76 (3.9, 15.1) **	

Note: The data are presented as % of participants in each sub-group of knowledge score tertiles and socio-demographics. Odds ratio (OR) and 95% confidence interval (95% CI) were calculated by running a regression analysis showing the odds of having a kale knowledge score in the highest tertile (tertile 3) compared to the lowest tertile (tertile 1) in all subjects, males only and females only. *p*-value less than 0.05 was considered significant. * and ** represent *p*-values at <0.05 and <0.01 levels, respectively.

**Table 4:** Kale consumption and its health effects in the study participants.

	All	Male	Female	*p*-Value
N	1200	476	724	
Have You Ever Heard about Kale?				
Yes	585 (48.8)	201 (42.2)	384 (53)	<0.001
No	615 (51.3)	275 (57.8)	340 (47)	
Have You Consumed Kale Before?				
Yes	276 (23)	87 (18.3)	189 (26.1)	0.002
No	924 (77)	389 (81.7)	535 (73.9)	
How Many Times a Week Were You Consuming Kale?				
Once	199 (72.1)	48 (55.2)	151 (79.9)	<0.001
2–3 times	61 (22.1)	33 (37.9)	28 (14.8)	
>3 times	16 (5.8)	6 (6.9)	10 (5.3)	
Have You Noticed Any Positive Effects on Health?				
Yes	178 (64.5)	62 (71.3)	115 (60.8)	0.11
No	98 (35.5)	25 (28.7)	74 (39.2)	
If Yes, Mention the Positive Effects?				
Weight Loss	91 (51.1)	28 (45.2)	63 (54.8)	0.14
Decrease in appetite	36 (20.2)	18 (29)	18 (15.7)	
Improved bowel movement	30 (16.9)	11 (17.7)	19 (16.5)	
Energy boost	21 (11.8)	5 (8.1)	15 (13)	
Any Side Effects?				
Yes	49 (17.8)	17 (19.5)	32 (16.9)	0.74
No	227 (82.2)	70 (80.5)	157 (83.1)	
If Yes, Mention the Side Effects?				
Constipation	25 (51)	10 (58.8)	15 (46.9)	0.69
Bloating	12 (24.5)	4 (23.5)	8 (25)	
Bowel irritability	10 (20.4)	2 (11.8)	8 (25)	
Others	2 (4.1)	1 (5.9)	1 (3.1)	
Mode of Consumption?				
Fresh	158 (57.2)	46 (52.9)	112 (59.3)	0.69
Cooked	53 (19.2)	17 (19.5)	36 (19)	
Supplement	47 (17)	18 (20.7)	29 (15.3)	
Powder	18 (6.5)	6 (6.9)	12 (6.3)	

Note: The data are presented as frequency (%) of participants in each sub-group. The difference between males and females was calculated by chi-squared test.

## Data Availability

Data are available from the corresponding author on reasonable request.

## Supplementary Materials

The following are available online at https://www.mdpi.com/article/10.3390/nu14020245/s1: Table S1. Kale knowledge questionnaire responses; Table S2. Average nutritional content of one cup of kale.
