# Supplementary material for: Awareness, Knowledge and Attitude towards ‘Superfood’ Kale and Its Health Benefits among Arab Adults
Source: Nutrients. 2022 Jan 7;14(2):245. doi: 10.3390/nu14020245 (PMC8782012; doi:10.3390/nu14020245)
Supplement: Supplementary file 1 [file nutrients-14-00245-s001.zip › nutrients-1465850-supplementary.pdf]

Awareness, Knowledge and Attitude towards Kale and Its Health Benefits According to the Socioeconomic Status in Saudi Adults

Hanan A. Alfawaz, Kaiser Wani, Haya Alrakayan, Abdullah M. Alnaami and Nasser M. Al-Daghri

Supplementary Table S1: Kale knowledge questionnaire responses.

	**All**	**Male**	**Female**	**p**
N	1200	476	724	
**Q1. Is Kale considered a high calorie food?**				
Yes	9 (0.8)	2 (0.4)	7 (0.9)	0.15
No	722 (60.2)	274 (57.6)	448 (61.9)	
Don't know	469 (39.1)	200 (42)	269 (37.2)	
**Q2. Is Kale considered a high nutritional value food?**				
Yes	761 (63.4)	285 (59.9)	476 (65.6)	0.11
No	11 (0.9)	4 (0.8)	7 (1)	
Don't know	428 (35.7)	187 (39.3)	241 (33.3)	
**Q3. Is Kale considered a food with antioxidant property?**				
Yes	358 (29.8)	128 (26.9)	230 (31.8)	0.07
No	401 (33.4)	147 (30.9)	254 (35.1)	
Don't know	441 (36.8)	201 (42.2)	240 (33.1)	
**Q4. Is Kale considered a food with immune boost property?**				
Yes	685 (57.1)	261 (54.8)	424 (58.6)	0.31
No	5 (0.4)	4 (0.8)	3 (0.4)	
Don't know	508 (42.3)	211 (44.3)	297 (41.0)	
**Q5. Does excessive eating of Kale cause constipation?**				
Yes	138 (11.5)	63 (13.2)	75 (10.4)	0.06
No	282 (23.5)	97 (20.4)	185 (25.5)	
Don't know	780 (65)	316 (66.4)	464 (64.1)	
**Q6. Is Kale considered a food rich in fibers?**				
Yes	663 (55.3)	242 (50.8)	421 (58.1)	**0.02**
No	17 (1.4)	5 (1.1)	12 (1.7)	
Don't know	520 (43.3)	229 (48.1)	291 (40.2)	
**Q7. Is Kale considered a food rich in Vitamin C?**				
Yes	374 (31.2)	138 (29)	236 (32.6)	0.37
No	111 (9.3)	43 (9)	68 (9.4)	
Don't know	715 (59.6)	295 (62)	420 (58.0)	
**Q8. Is Kale consumption considered bad for people suffering from digestive problems?**				

Yes	164 (13.7)	76 (16)	88 (12.2)	0.05
No	236 (19.7)	81 (17)	155 (21.4)	
Don't know	800 (66.7)	319 (67)	481 (66.4)	
**Q9. Is Kale consumption considered a food with anti-carcinogenic properties?**				
Yes	206 (17.2)	80 (16.8)	126 (17.4)	0.88
No	115 (9.6)	48 (10.1)	67 (9.3)	
Don't know	879 (73.3)	348 (73.1)	531 (73.3)	
**Q10. Is Kale consumption considered a food with anti-inflammatory properties?**				
Yes	228 (19)	92 (19.3)	136 (18.8)	0.92
No	90 (7.5)	37 (7.8)	53 (7.3)	
Don't know	882 (73.5)	347 (72.9)	535 (73.9)	
**Q11. Is Kale consumption considered a food high in fats?**				
Yes	22 (1.8)	5 (1.1)	17 (2.3)	0.19
No	693 (57.8)	271 (56.9)	422 (58.3)	
Don't know	485 (40.4)	200 (42)	285 (39.4)	
**Q12. Does Kale play a role in controlling chronic diseases?**				
Yes	358 (29.8)	151 (31.7)	207 (28.6)	0.47
No	50 (4.2)	18 (3.8)	32 (4.4)	
Don't know	792 (66)	307 (64.5)	485 (67.0)	
**Q13. Does the nutritional value of Kale increase after cooking?**				
Yes	303 (25.3)	142 (29.8)	161 (22.2)	**<0.001**
No	193 (16.1)	55 (11.6)	138 (19.1)	
Don't know	704 (58.7)	279 (58.6)	425 (58.7)	

Note: The data was presented as frequency (%).The difference between males and females was calculated by chi-squared test.

Supplementary Table S2. Average nutritional content of one cup of kale.

		Amount in 1 cup	Average RDI	Average RDI satisfied (%)
energy	(kcal)	49	2400	2.04
		Macronutrients		
Carbohydrate	(g)	8.75	130	6.73
Dietary Fibers	(g)	3.6	28	12.86
protein	(g)	4.28	51	8.39
Total fats	(g)	0.93	65	1.43
		Minerals		
Ca	(mg)	150	1100	13.64
Fe	(mg)	1.47	13	11.31
Mg	(mg)	47	370	12.7
P	(mg)	92	700	13.14
K	(mg)	491	4700	10.45
Na	(mg)	38	1400	2.71
Zn	(mg)	0.56	9.5	5.89
Cu	(mg)	0.8	900	0.09
Mn	(mg)	0.6	2.05	29.27
		Vitamins		
Vitamin C	(mg)	21	82.5	25.45
thiamin	(mg)	0.11	1.15	9.57
riboflavin	(mg)	0.13	1.15	11.3
niacin	(mg)	1	15	6.67
B-6	(mg)	0.271	1.5	18.07
folate	(mg)	141	400	35.25
Vitamin A	(mg)	172	1150	14.96
Vitamin E	(mg)	1.54	15	10.27
Vitamin K	(mg)	494	150	329.33

Note: The data was drawn from USDA website available at https://ndb.nal.usda.gov/ndb. The nutritional requirements are estimated for an average adult and may vary according to age and sex.
